# Biosynthesis of 3‑Hydroxy-3-methylbutyrate by Probiotic *Escherichia coli* Nissle 1917

**DOI:** 10.1021/acs.jafc.6c08735

**Published:** 2026-09-08

**Authors:** Jenny Ji-Chan Hung, Tina Wen-Ting Shih, Alice Pin-Yi Du, Ethan I. Lan

**Affiliations:** †Ph.D. Degree Program of Biomedical Science and Engineering, ‡Institute of Molecular Medicine and Bioengineering, §Department of Biological Science and Technology, 34914 National Yang Ming Chiao Tung University, Hsinchu City 300, Taiwan

**Keywords:** metabolic engineering, 3-Hydroxy-3-methylbutyrate, 3-hydroxyisovalerate, Escherichia coli Nissle 1917, pantothenic acid, vitamin B5

## Abstract

3-Hydroxy-3-methylbutyrate (HMB) is an important nutritional supplement for managing sarcopenia. This study engineered an HMB biosynthetic pathway in the probiotic *Escherichia coli* Nissle 1917 (EcN). The initial engineered strain exhibited low HMB titers, as carbon flux was primarily diverted toward acetate formation. Deleting the pyruvate oxidase gene (*poxB*) reduced acetate but caused significant pyruvate accumulation, revealing a severe kinetic bottleneck in endogenous pyruvate dehydrogenase (PDH) activity. Supplementing pantothenic acid (Vitamin B5) profoundly expanded the intracellular coenzyme A (CoA) pool, providing the essential backbone for pathway intermediates. This strategy enhanced PDH flux and improved HMB production by 5.5-fold. Through fed-batch fermentation, the engineered strain achieved an HMB titer of 8.6 g/L with a yield of 0.14 g/g glucose. The results of this study provided a probiotic strain that can synthesize HMB *de novo* and demonstrated the importance of pantothenic acid availability for EcN metabolic engineering.

## Graphical abstract

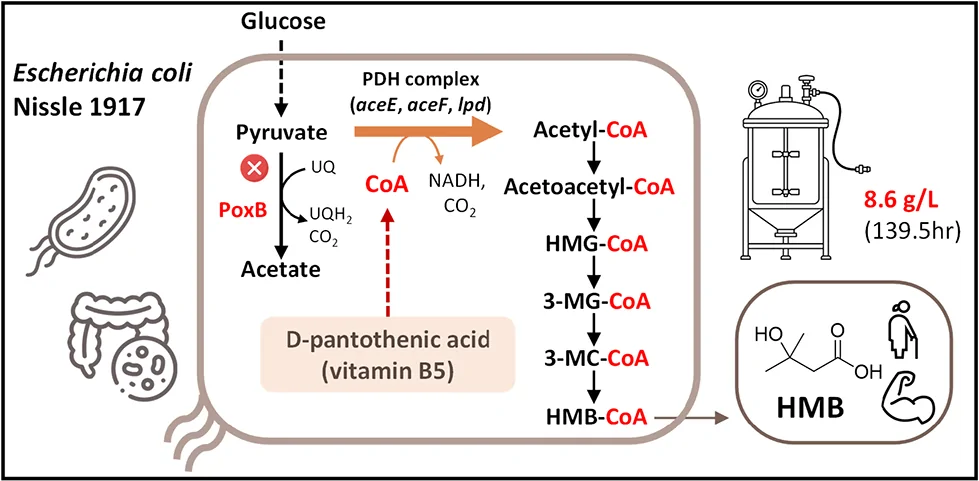

## Introduction

With the rapid advancement of medical technology, global life expectancy is increasing, leading to a significant shift in demographic structures. According to the United Nations, one in six people worldwide will be aged 65 or older by year 2050.^1^ This aging process is accompanied by a progressive loss of physiological integrity, resulting in impaired body functions and an increased risk of chronic diseases.^2^ Among these, sarcopenia is an age-related decline in muscle mass and strength and affects up to 16% of the elderly population globally.^3^ Sarcopenia leads to impaired mobility, reduced quality of daily living, and a higher risk of falls.^4^ Muscle weakness is estimated to account for billions in incremental healthcare expenditure annually.^5,6^ In recent years, 3-hydroxy-3-methylbutyrate (HMB), also known as 3-hydroxyisovalerate, has been increasingly used as a dietary supplement for sarcopenia management and by individuals engaged in fitness training.^7–11^ As a catabolite of leucine, HMB stimulates muscle protein synthesis via the mTOR pathway while simultaneously inhibiting protein degradation.^11^ A daily dosage of approximately 3 g can effectively stimulate muscle repair.^8,12,13^ However, HMB levels converted from dietary leucine are insufficient to achieve this therapeutic threshold. Consequently, the demand for oral HMB supplements has surged, with the global HMB market reaching hundreds of millions of dollars.^14^ Leading nutritional companies, such as Abbott, have already commercialized HMB-enriched products like Ensure and Abound to address these clinical and consumer needs.^15^

HMB is currently produced from chemical synthesis that relies on the use of toxic and nonrenewable resources, making biotransformation a more sustainable alternative.^16,17^ Early biological approaches used yeast *Galactomyces reessii* to produce HMB from 3-methylbutyric acid or metabolically engineered *Escherichia coli* to produce HMB from leucine, both of which are relatively expensive substrates.^18–20^ In our previous work, we established a *de novo* HMB biosynthetic pathway in *E. coli* BL21­(DE3), enabling direct production of HMB from glucose and achieving a titer of 17.7 g/L.^21^ This result demonstrated the feasibility of microbial HMB production using renewable resources. Although commercial purification processes such as anion-exchange chromatography, ultrafiltration, and Triton X phase separation are routinely employed to remove contaminating lipopolysaccharide (LPS) produced by *E. coli* BL21­(DE3), these methods add manufacturing costs.^22^ Furthermore, the resulting products may contain residual endotoxin levels sufficient to trigger adverse immune responses or a pyrogenic effect.^23,24^

To overcome these limitations, *E. coli* Nissle 1917 (EcN) is an attractive alternative host for HMB bioproduction. EcN is a clinically recognized probiotic with an established safety record, exhibiting only 0.86% of the LPS immunotoxicity found in strain BL21­(DE3).^25^ Furthermore, it possesses numerous anti-inflammatory properties that help optimize the intestinal microbiota.^26–28^ While many probiotic species are also excellent therapeutic candidates, the advanced genetic tools and well-established cultivation of EcN enhance its potential as a microbial factory.^29–32^ Moreover, because EcN shares a highly conserved genetic background with laboratory *E. coli* strains, the HMB biosynthetic pathway and genetic elements previously established can be adapted with minimal re-engineering. The engineered pathway is also well suited to the *E. coli* cellular environment, as two key enzymes (AtoB and YciA) are endogenous to *E. coli*, and the average GC content of the heterologous genes closely matches that of the *E. coli* genome (approximately 50%). In contrast, probiotic hosts such as *Bacillus subtilis* and *Lactobacillus* generally possess lower genomic GC contents (approximately 30%), which may reduce heterologous gene expression and mRNA stability. These genetic and metabolic advantages, together with EcN’s established safety profile and potential as a live biotherapeutic chassis, make it an attractive platform for developing engineered probiotics for HMB production and delivery. Therefore, this study aimed to develop an HMB-producing EcN strain by introducing a *de novo* biosynthetic pathway.

## Materials and Methods

### Strains and Plasmids Construction

The bacterial strains and plasmids utilized in this study are summarized in Table 1. The synthetic HMB biosynthetic pathway genes were polycistronically expressed under the control of the isopropyl β-D-1-thiogalactopyranoside (IPTG)-inducible P_LlacO1_ promoter. Each gene was preceded by a synthetic ribosome binding site (RBS) with the sequence 5′-AGGAGATATACC-3′ to ensure efficient translation initiation. Plasmids were constructed via the Gibson assembly method^33^ and propagated in *E. coli* XL1-blue for cloning and maintenance. The core HMB production plasmids (pML97, pML98, pML100, pML101, and pRM27) were obtained from our previous study.^21^ All newly constructed plasmids, including pJH200 (encoding the *aceEF-lpd* operon of EcN) and pJH203 (encoding *acs*), were verified by sequencing. Chromosomal gene deletions in *E. coli* Nissle 1917 were achieved using P1 phage transduction^34^ with donor strains from the Keio collection.^35^ All genomic modifications were confirmed through colony PCR.

**Table 1:** Strains and Plasmids Used in This Study

**strain**	**genotype**	reference
**EcN**	*E. coli Nissle 1917* O6:K5:H1	Mutaflor
**XL1-blue**	*recA1 endA1 gyrA96 thi-1 hsdR17 supE44 relA1 lac* [F’ *proAB lacI* ^*q*^ *Z*Δ*M15 Tn10* (Tet^r^)]	laboratory collection
**TS06**	EcN Δ*pta*	this study
**TS07**	EcN Δ*poxB*	this study
**plasmid**	**genotype**	**Reference**
**pML97**	P_LlacO1_::*atoB, mvaS, liuC, aibA, aibB, yciA*; ColE1 ori; Amp^R^	^21^
**pML98**	P_LlacO1_::*atoB, mvaS, liuC, aibA, aibB, yciA*; ColA ori; Kan^R^	^21^
**pML100**	P_LlacO1_::*atoB, mvaS, liuC, aibA, aibB, yciA*; pSC101 ori; Spec^R^	^21^
**pML101**	P_LlacO1_::*atoB, mvaS, liuC, aibA, aibB, yciA*; CloDF ori; Kan^R^	^21^
**pRM27**	P_LlacO1_::*atoB, mvaS, liuC, aibA, aibB, yciA*; RSF1030 ori; Kan^R^	^21^
**pJH203**	P_LlacO1_::*acs*; pSC101 ori; Spec^R^	this study
**pJH200**	P_LlacO1_::*aceE, aceF, lpd*; pSC101 ori; Spec^R^	this study

### Mediums and Culture Conditions

All chemicals and reagents were purchased from commercial suppliers and used as received. Specifically, Na_2_HPO_4_·7H_2_O, KH_2_PO_4_, thiamine (Vitamin B1), and glucose were obtained from Sigma-Aldrich (St. Louis, MO, USA). NaCl and NH_4_Cl were sourced from Fisher Scientific (Waltham, MA, USA) and Macron Fine Chemicals (Center Valley, PA, USA), respectively. Calcium chloride CaCl_2_ was purchased from Amresco (Solon, OH, USA), and the sodium salt of Vitamin B5 was supplied by Thermo Fisher Scientific (Waltham, MA, USA). Luria–Bertani (LB) medium and SOC medium components were purchased from FocusBio (Taiwan).

For routine cloning and seed culture preparation, *E. coli* strains were cultivated in LB medium at 37 °C with rotary shaking at 250 rpm. SOC medium was utilized for cell recovery following electroporation. For HMB production, M9 minimal medium was employed, containing (per liter): 3 g KH_2_PO_4_, 0.5 g NaCl, 12.8 g Na_2_HPO_4_·7H_2_O, 1 mM MgSO_4_, 0.5 g NH_4_Cl, 0.1 mM CaCl_2_, 100 mg vitamin B1 and 20 g glucose.^36^ Vitamin B5 supplementation was performed using a concentrated stock solution of D-pantothenic acid sodium salt. Antibiotics were added as required at the following concentrations: 50 μg/mL kanamycin, 100 μg/mL ampicillin and 100 μg/mL spectinomycin.

### HMB Production and Product Quantification

Production strains were prepared by transforming the respective plasmids into the host strains via electroporation. Seed cultures were prepared by inoculating individual colonies into 2 mL of LB medium and cultivating them overnight at 37 °C. For HMB production, the seed cultures were inoculated at a 1% (V/V) ratio into 2 mL of M9 production medium in test tubes. The cultures grew at 37 °C with rotary shaking at 250 rpm.

When the optical density reached OD_600_ 0.4–0.6, protein expression was induced by the addition of 0.1 mM IPTG. After 24 h of postinduction cultivation, samples were harvested to determine cell growth and metabolite concentrations. Cell density was measured as OD_600_ using a spectrophotometer. For metabolite quantification, the cultures were centrifuged at 18,000*g* for 5 min, and the supernatants were analyzed by high-performance liquid chromatography (HPLC) Shimadzu LC-2030C equipped with photodiode array detector and refractive index detector. Metabolites, including HMB, glucose, and organic acid byproducts, were separated using a Bio-Rad Aminex HPX-87H HPLC organic acid column (300 × 7.8 mm, 9 μm) at 35 °C. with a mobile phase of 5 mM H_2_SO_4_ at a flow rate of 0.6 mL/min. For each analysis, 20 μL of the sample was injected.

### Metabolic Profiling of Intracellular CoA Species

For the analysis of intracellular metabolites, cultivation was performed in 50 mL of M9 medium using flasks. The inoculation ratio, induction timing, and environmental condition were maintained identical to the test tube scale production. After 24 h of cultivation, the 50 mL cultures were harvested via centrifugation at 4 °C. The resulting cell pellet was immediately resuspended in 1 mL of a precooled extraction solvent (methanol:acetonitrile:water = 2:2:1, v/v/v). To ensure comprehensive metabolite release, the suspension was vortexed for 1 min and subjected to three freeze–thaw cycles (−80 °C, 15 min). Following extraction, the mixture was centrifuged to remove cellular debris, and the supernatant was recovered. The sample was further purified by deproteinization using Nanosep Centrifugal Devices equipped with a 10 K Omega Membrane (Pall OD010C35) prior to LC-MS/MS analysis.

Intracellular CoA species were analyzed using a Shimadzu LC-MS 8060 triple quadrupole mass spectrometer coupled with a YMC-Triart C18 ExRS column (150 × 2.1 mm, 1.9 μm). The mobile phase and gradient conditions were adapted from a previously established protocol with minor adjustments.^37^ A binary gradient system was employed at a flow rate of 0.3 mL/min, comprising buffer A (10 mM tributylamine, 15 mM acetic acid, and 0.4 mM NH_4_F) and buffer B (100% methanol with 0.4 mM NH_4_F). The elution profile was programmed as follows: 0 min, 5% B; 0–4 min, linear increase to 25% B; 4–10 min, linear increase to 98% B; 10–20 min, held at 98% B; and 20–22 min, returned to 5% B.

Metabolites were detected in negative multiple reaction monitoring (MRM) mode with the following instrument settings: capillary voltage 4000 V, fragmentor voltage 135 V, and gas flow rates of 3 L/min (nebulizing), 15 L/min (heating), and 5 L/min (drying). The column temperature was set at 50 °C, and 10 μL of sample was injected per run. The specific MRM transitions utilized were: acetyl-CoA (808.00 > 408.00), CoA (766.05 > 408.00), acetoacetyl-CoA (850.00 > 426.00), HMB-CoA (866.00 > 408.00), 3-MG-CoA (892.00 > 408.00), 3-MC-CoA (848.00 > 408.00), and HMG-CoA (910.00 > 408.00). The identity of each intracellular metabolite was rigorously confirmed by comparing its mass-to-charge (*m*/*z*) ratio and fragmentation patterns with those of commercial standards. Since HMB-CoA and 3-MG-CoA are not commercially available, standards were generated via enzymatic synthesis. HMB-CoA was synthesized in a 30 min reaction mixture containing 30 mM HMB, 1 mM CoA, 5 mM ATP, 0.1 mM MgCl_2_, 1 mM DTT, and 20 μg of acyl-CoA synthetase Acs. Similarly, 3-MG-CoA was produced by incubating 1 mM HMG-CoA with 20 μg of LiuC and 1 mM DTT for 30 min. The resulting reaction products were used as qualitative references to validate the characteristic MRM transitions and retention times for the corresponding target peaks in the biological samples. Relative metabolite levels were determined by normalizing the peak area signals to the dry cell weight (DCW) of each sample. Final values are expressed as a ratio relative to the control strain, which was assigned a value of 1.0 to facilitate comparative analysis.

### Microbial Fermentation

Fed-batch fermentation was performed in a 5-L bioreactor (Biostat A; Sartorius, Göttingen, Germany) with an initial working volume of 2 L. The fermentation medium consisted of M9 minimal medium supplemented with 40 g/L glucose and 100 mg/L Vitamin B5. Seed cultures were prepared by inoculating single colonies into 4 mL of LB medium and cultivating them overnight at 37 °C. A 2.5% (V/V) aliquot of the overnight culture was then transferred into 100 mL of M9 bioreactor medium in a baffled flask and incubated at 37 °C and 250 rpm for 12–14 h before being used to inoculate the bioreactor to an initial OD_600_ = 0.1. The culture pH was maintained at 7.0 via the automatic addition of 2N NaOH, and the dissolved oxygen (DO) level was kept at 30% of air saturation. The temperature was initially set at 37 °C. Upon reaching an OD_600_ of 1.0, protein expression was induced by the addition of 0.1 mM IPTG, and the temperature was subsequently shifted to 30 °C to facilitate HMB biosynthesis. A fed-batch feeding strategy was implemented using a solution containing M9 medium components, 50% (W/V) glucose, 0.1 mM IPTG, and 50 μL/mL kanamycin. Furthermore, a 0.02 M (NH_4_)_2_SO_4_ solution was fed every 12 h until the culture reached the stationary phase. To maximize carbon conversion efficiency, a strategic glucose starvation phase was implemented during the late stage of cultivation.

## Results and Discussion

### Construction of *De Novo* HMB Biosynthetic Pathway in *E. coli* Nissle 1917

The *de novo* HMB biosynthetic pathway previously established^21^ was introduced into EcN. This pathway facilitates the conversion of acetyl-CoA to HMB through five enzymatic steps: (1) the condensation of two acetyl-CoA molecules into acetoacetyl-CoA by acetoacetyl-CoA thiolase (AtoB from *E. coli*); (2) the synthesis of 3-hydroxymethylglutaryl-CoA (HMG-CoA) by HMG-CoA synthase (MvaS from *Staphylococcus aureus*); (3) the dehydration of HMG-CoA into 3-methylglutaconyl-CoA (3-MG-CoA) by 3-methylglutaconyl-CoA hydratase (LiuC from *Myxococcus xanthus*); (4) the decarboxylation of 3-MG-CoA to 3-methylcrotonyl-CoA (3-MC-CoA) by glutaconyl-CoA decarboxylase (AibAB from *M. xanthus*); and (5) the hydration of 3-MC-CoA to HMB-CoA by LiuC, followed by thioesterase-mediated hydrolysis (YciA from *E. coli*) to liberate the final product, HMB (Figure 1).

**Figure 1:**
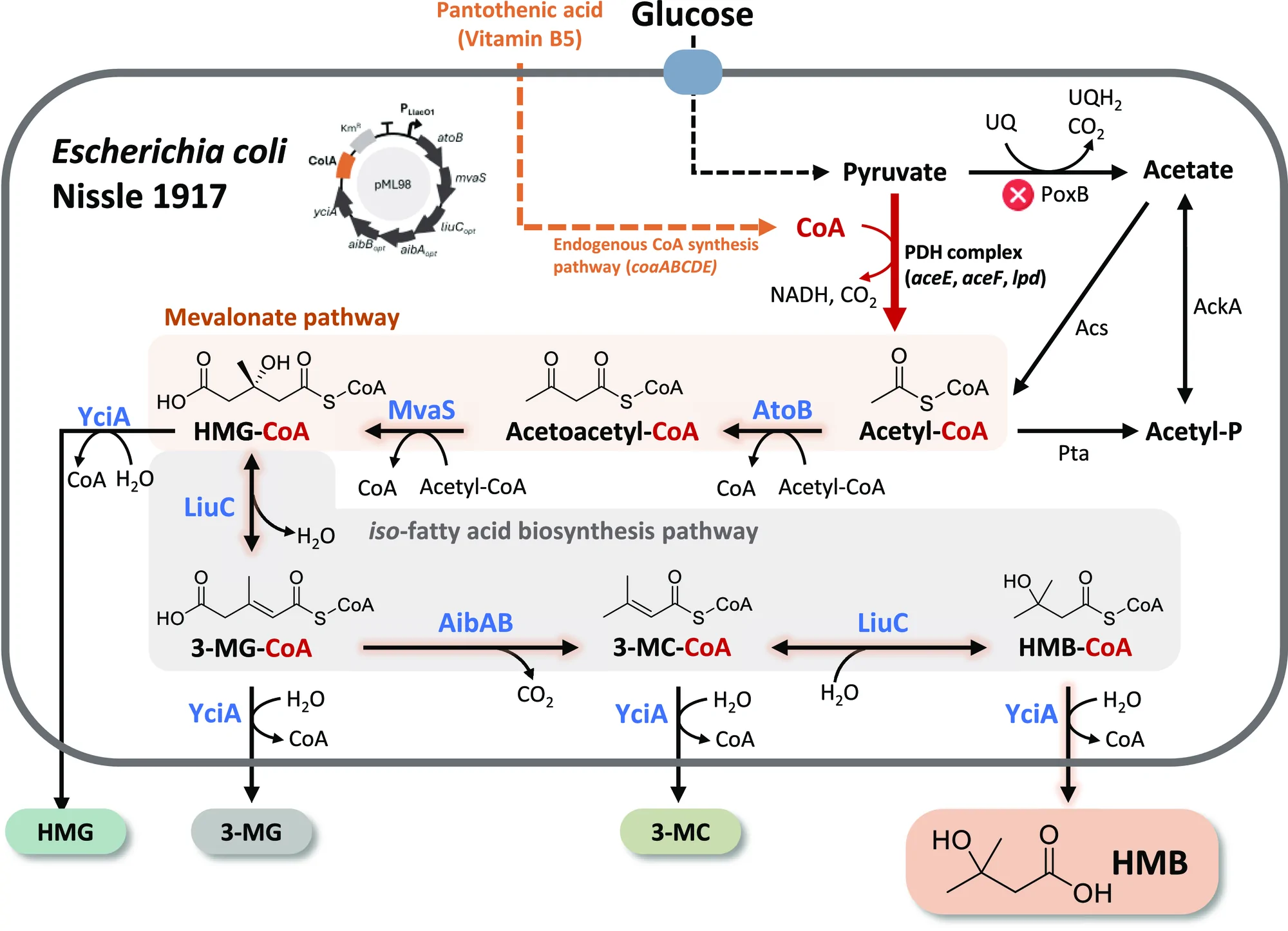
HMB production in engineered *Escherichia coli* Nissle 1917. The engineered pathway utilizes acetyl-CoA to synthesize HMB-CoA through a cascade of reactions catalyzed by acetoacetyl-CoA thiolase (AtoB) from *Escherichia coli*, HMG-CoA synthase (MvaS) from *Staphylococcus aureus*, and 3-methylglutaconyl-CoA hydratase (LiuC) and glutaconyl-CoA decarboxylase (AibAB) from *Myxococcus xanthus*. HMB is subsequently generated via the thioesterase-mediated hydrolysis of HMB-CoA by acyl-CoA thioesterase (YciA) from *E. coli*. Nonspecific hydrolysis of the CoA-bound intermediates by YciA also leads to the formation of organic acid byproducts, including HMG, 3MG, and 3MC. Key endogenous metabolic nodes from the host strain dictating carbon flux are illustrated, including the pyruvate dehydrogenase complex (AceEF-Lpd) for acetyl-CoA supply, competing acetate-forming routes via pyruvate oxidase (PoxB) and acetate kinase (AckA), and the acetate assimilation pathway mediated by acetyl-CoA synthetase (Acs). Additionally, the endogenous biosynthetic pathway converting pantothenic acid into CoA is depicted to highlight the mechanism of intracellular CoA pool expansion.

Given that plasmid maintenance and copy number affect genes and their corresponding enzyme expression, five expression vectors harboring different origins of replication—pSC101, ColA, CloDF, ColE1, and RSF—were evaluated for expressing HMB biosynthetic pathway in EcN. As shown in Figure 2A, HMB was successfully produced by all recombinant EcN strains. Among the evaluated vectors, the vector with ColA origin enabled the highest production of HMB with a titer of 0.34 g/L in 24 h. Thioester-hydrolyzed products of pathway intermediates, including 3-hydroxymethylglutarate (HMG), 3-methylglutaconate (3MG), and 3-methylcrotonate (3MC), were also detected. This result is likely due to the broad substrate specificity of the thioesterase YciA.^21^ Compared to the 2.4 g/L of HMB produced by strain BL21­(DE3)^21^ using the same plasmid, the HMB production performance in EcN was significantly lower. Correspondingly, strain EcN produced 2.6 g/L of acetate whereas strain BL21­(DE3) only produced 0.28 g/L. This high level of acetate secretion suggested a significant diversion of acetyl-CoA flux, identifying a critical challenge to using EcN for the synthesis of HMB. Interestingly, the plasmid pML97 using high copy number ColE1 origin exhibited poor and unstable performance. This is potentially attributed to plasmid incompatibility as EcN naturally harbors two endogenous plasmids with ColE1-like replication mechanisms.^38^ These results show the necessity of host-specific engineering to minimize byproduct competition in EcN.

**Figure 2:**
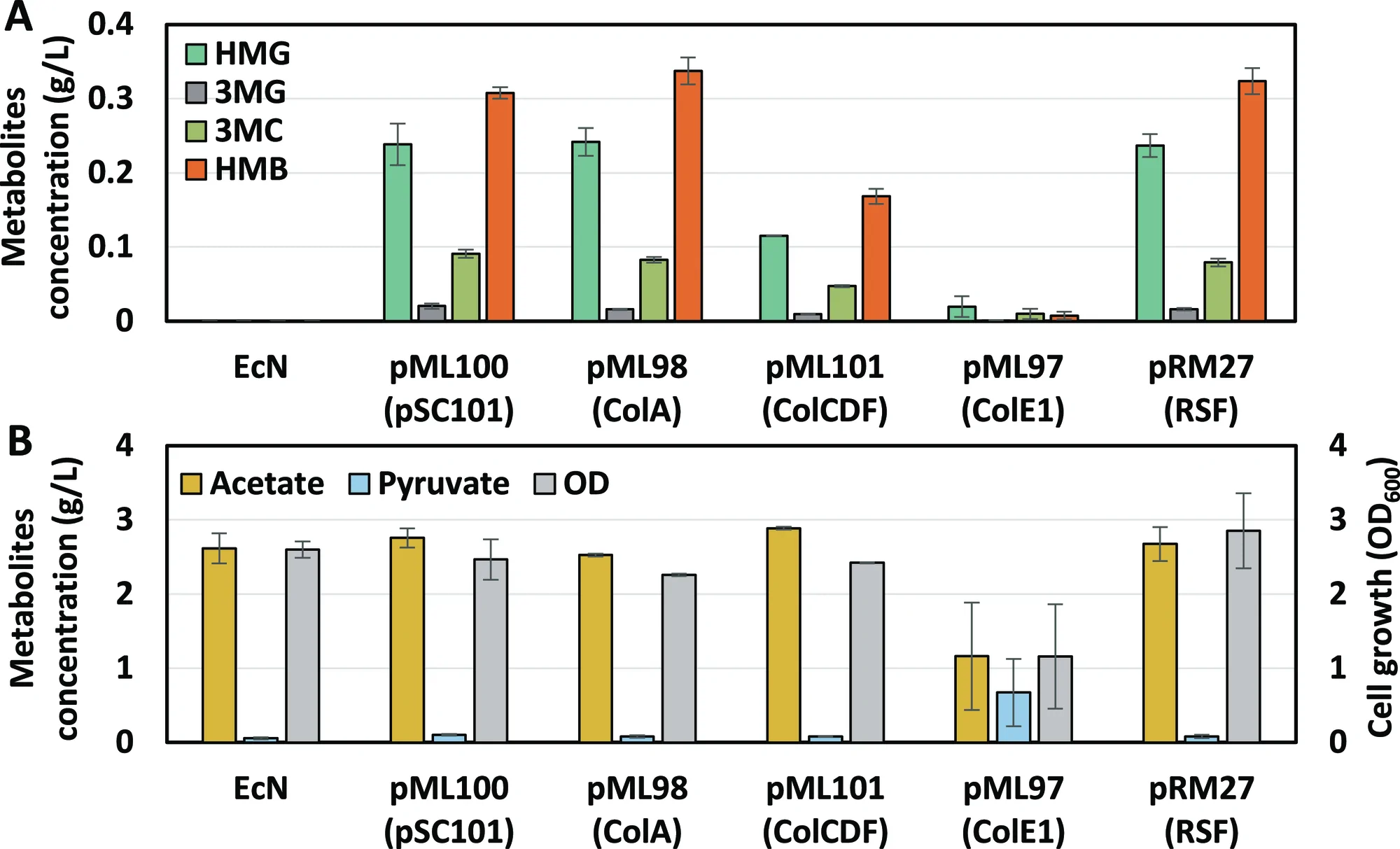
Cultivation results of the recombinant *E. coli* Nissle 1917 strains producing HMB. (A) Titers of HMB and pathway intermediates (HMG, 3MG, and 3MC) after 24 h of cultivation. (B) Concentrations of major byproducts (acetate and pyruvate) and cell growth. The synthetic HMB pathway was expressed using various replication origins: pSC101 (pML100), ColA (pML98), CloDF (pML101), ColE1 (pML97), and RSF (pRM27). Data are presented as the mean ± standard deviation of triplicate experiments (*n* = 3).

### Genetic Approaches for Minimizing Acetate Accumulation

To reassimilate the acetate produced, we first overexpressed acetyl-CoA synthetase (Acs) together with HMB biosynthesis pathway in strain EcN (Figure 3A). As the results shown in Figure 3B, overexpressing *acs* (strain EcN harboring plasmids pML98 and pJH203) led to a slight improvement in HMB production, reaching 0.58 g/L. However, acetate levels remained substantial at approximately 2.3 g/L, suggesting that the rate of acetate reassimilation could not sufficiently compensate for its high rate of formation in EcN.

**Figure 3:**
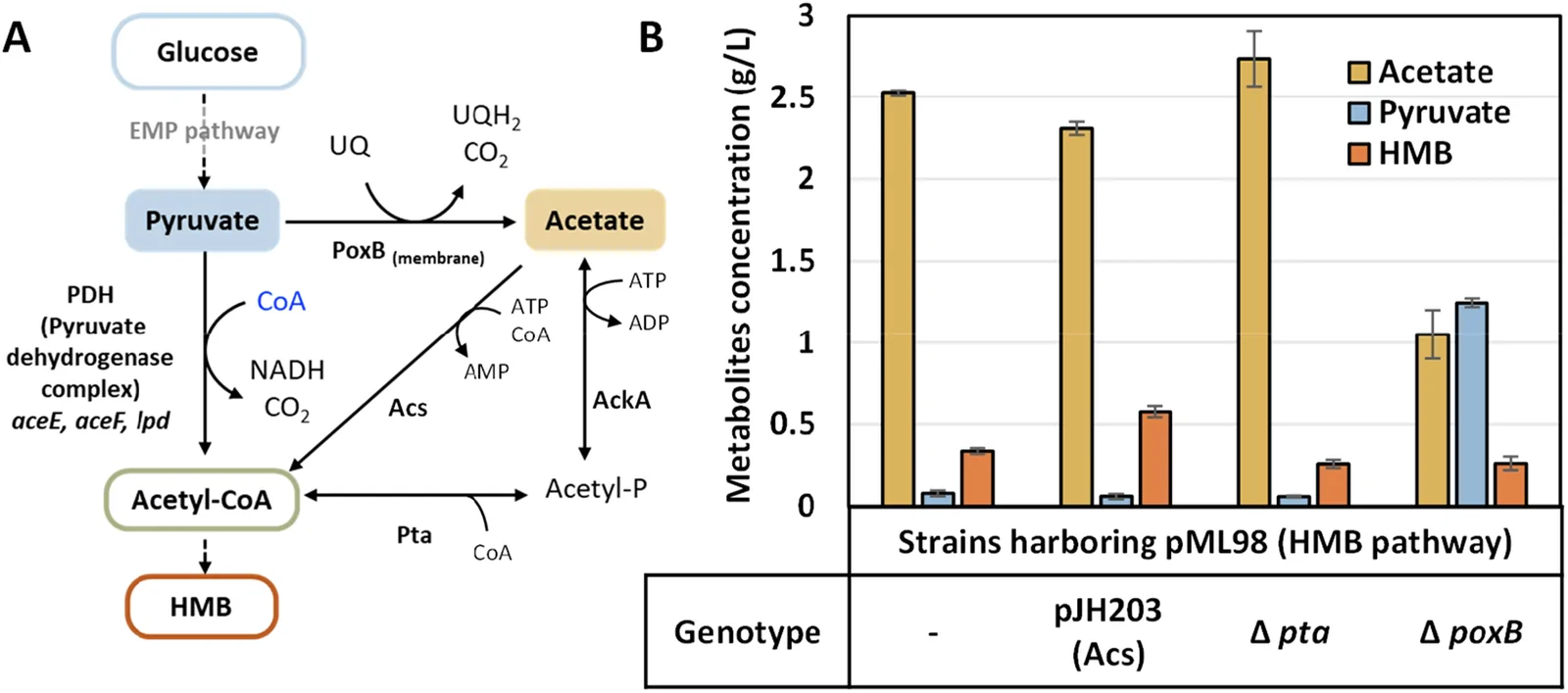
Eliminating main byproduct acetate accumulation by overexpressing acetate assimilation pathway or knocking out competing pathways. Production profiles of HMB and its byproducts in strains transformed with the HMB pathway plasmid pML98, either cotransformed with the Acs-overexpressing plasmid pJH203 or introduced into the Δ*pta* (TS06) and Δ*poxB* (TS07) strains in 24 h. (A) Schematic of HMB competing pathways. (B) Concentration of HMB, acetate, pyruvate. Data are presented as the mean ± standard deviation of triplicate experiments (*n* = 3).

To eliminate the substantial acetate accumulation, we knocked out the primary metabolic routes responsible for acetate formation. In *E. coli*, acetate is primarily synthesized through two pathways: the pyruvate oxidase pathway (PoxB), which directly converts pyruvate to acetate, and the phosphotransacetylase-acetate kinase pathway (Pta-AckA), which converts acetyl-CoA to acetate (Figure 3A). The deletion of *pta* (strain TS06) showed no significant effect on reducing acetate secretion, implying that the Pta-AckA pathway is not the predominant source of acetate in strain EcN under the tested conditions. In contrast, the knockout of *poxB* (strain TS07) was effective, reducing the acetate titer from 2.6 to 1.1 g/L (Figure 3B). This result indicated that PoxB-mediated shunt from pyruvate is a major source of acetate. Compared to our previous result that showed *poxB* deletion was ineffective for reducing acetate secretion in BL21­(DE3), this finding showed a significant difference between the strains EcN and BL21­(DE3) at the pyruvate-acetate nodes. However, upon reduction of acetate secretion, 1.2 g/L pyruvate was secreted into culture medium instead. This result indicated a potential limitation in the endogenous PDH activity of EcN which appears insufficient to accommodate the increased pyruvate load. Consequently, enhancing the flux through PDH was identified as the next step to drive the accumulated carbon flux effectively toward the HMB biosynthetic pathway.

### Vitamin B5 Supplementation as a Key Strategy to Enhance HMB Biosynthesis

After identifying the possible kinetic limitation at the pyruvate-to-acetyl-CoA node, we first attempted to enhance the flux by overexpressing the *aceEF-lpd* operon which codes for the endogenous PDH complex. Concurrently, the supplementation of various PDH-related cofactors was also evaluated, including thiamine (B1) for thiamine pyrophosphate (TPP), riboflavin (B2) for FAD, lipoic acid, and pantothenic acid (B5) for Coenzyme A (CoA) (Figure 4A).

**Figure 4:**
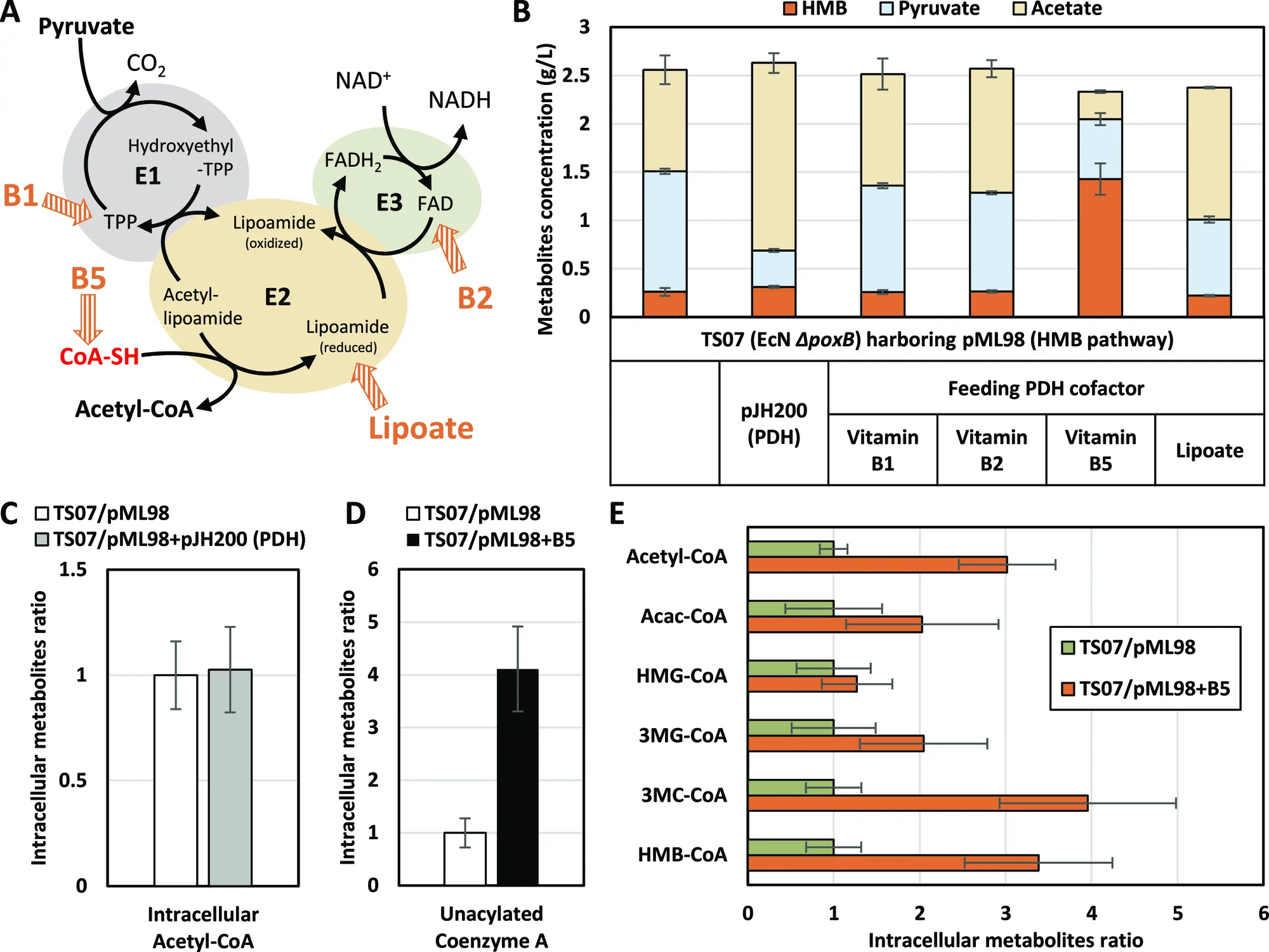
Effect of enhancing intracellular PDH activity through genetic engineering and cofactor supplementation. (A) Schematic of PDH enzyme mechanism and the functional role of the supplemented cofactors. (B) Production profiles of HMB and its byproducts in strain TS07 (EcN ΔpoxB) harboring the HMB pathway plasmid pML98 after 24 h. The strain was either cotransformed with the PDH-overexpressing plasmid pJH200 or supplemented with 100 mg/L of PDH-related cofactors (Vitamin B1, B2, B5, or lipoate). (C) Intracellular Acetyl-CoA concentration ratios. (D) Intracellular nonesterified coenzyme A (unacylated-CoA) concentration ratios. (E) Intracellular concentration ratios of CoA species associated with the HMB-CoA biosynthetic pathway. The signal areas were normalized to dry cell weight (DCW) and expressed as fold changes relative to the TS07/pML98 control strain (set as 1.0). Data are presented as the mean ± standard deviation of triplicate experiments (*n* = 3).

Experimental results showed that while overexpressing the endogenous PDH complex successfully reduced the extracellular accumulation of pyruvate from 1.2 to 0.38 g/L, it failed to improve the HMB titer (Figure 4B). Instead, the redirected carbon flux resulted in a resurgence of acetate accumulation. To elucidate this phenomenon, we quantified the intracellular acetyl-CoA levels using LC-MS/MS. The analysis revealed that the acetyl-CoA pool did not meaningfully increase in the PDH-overexpressing strain (Figure 4C). This suggests that merely elevating the PDH enzyme level is insufficient to drive the flux toward HMB biosynthesis. Without a proportional increase in cofactor availability or a strong downstream metabolic pull, the rapidly generated acetyl-CoA likely overflows back into acetate-producing pathways.

Surprisingly, among the evaluated cofactors supplementation, adding pantothenic acid triggered a metabolic shift. The addition of pantothenic acid led to an approximately 5.5-fold increase in HMB production by strain TS07 harboring plasmid pML98, rising from 0.26 to 1.4 g/L within 24 h. Pantothenic acid is a direct metabolic precursor of coenzyme A (CoA). Following cellular uptake, pantothenic acid is converted to CoA through the endogenous *coaABCDE* biosynthetic pathway, in which pantothenate kinase (CoaA), a key regulatory enzyme in CoA biosynthesis, catalyzes the first committed step.^39^ Its supplementation has been documented as a strategy to expand the intracellular CoA pool.^40,41^ LC-MS/MS metabolic profiling showed a significant increase in the intracellular coenzyme A concentration in the B5-supplemented group (Figure 4D). Consequently, the relative concentrations of CoA-bound intermediates of the HMB biosynthetic pathway were also substantially elevated (Figure 4E). These findings indicated that the endogenous CoA pool in EcN was originally a limiting factor. The expansion of the free CoA pool not only fulfilled the cofactor requirement for PDH activity but also provided the necessary backbone for the CoA-dependent intermediates for HMB biosynthesis. This increased concentration of the pathway intermediates accelerated the overall downstream reaction rate, effectively creating a strong driving force that pushes the carbon flux into the synthetic HMB pathway. This efficient redirection of acetyl-CoA prevented its overflow into acetate and rapidly consumed the upstream pyruvate, potentially explaining the concomitant decrease in both byproduct levels.

### HMB Production in Fed-Batch Fermentation

To evaluate the scalability and industrial potential of the engineered EcN strain TS07 (ΔpoxB)/pML98, fed-batch fermentation was conducted in a 5-L bioreactor using minimal M9 medium supplemented with glucose. The fermentation broth was supplemented with 100 mg/L pantothenic acid to enhance HMB titer. During the fermentation, the engineered strain demonstrated steady HMB accumulation (Figure 5A). To optimize carbon conversion efficiency, a glucose starvation strategy was implemented^42,43^ (Figure 5B). By intentionally restricting glucose feeding, we induced a carbon-limited environment that triggered the reassimilation of the secreted acetate. Notably, this recycled acetate was able to redirect back into the acetyl-CoA pool, allowing HMB production to steadily increase without stagnation (Figure 5A). After 139.5 h, the engineered strain achieved a final HMB titer of 8.6 g/L, with an overall productivity of 0.061 g/L/h and a conversion yield of 0.14 g/g glucose, representing approximately 34% of the maximum theoretical yield (0.44 g/g) (Table 2).

**Figure 5:**
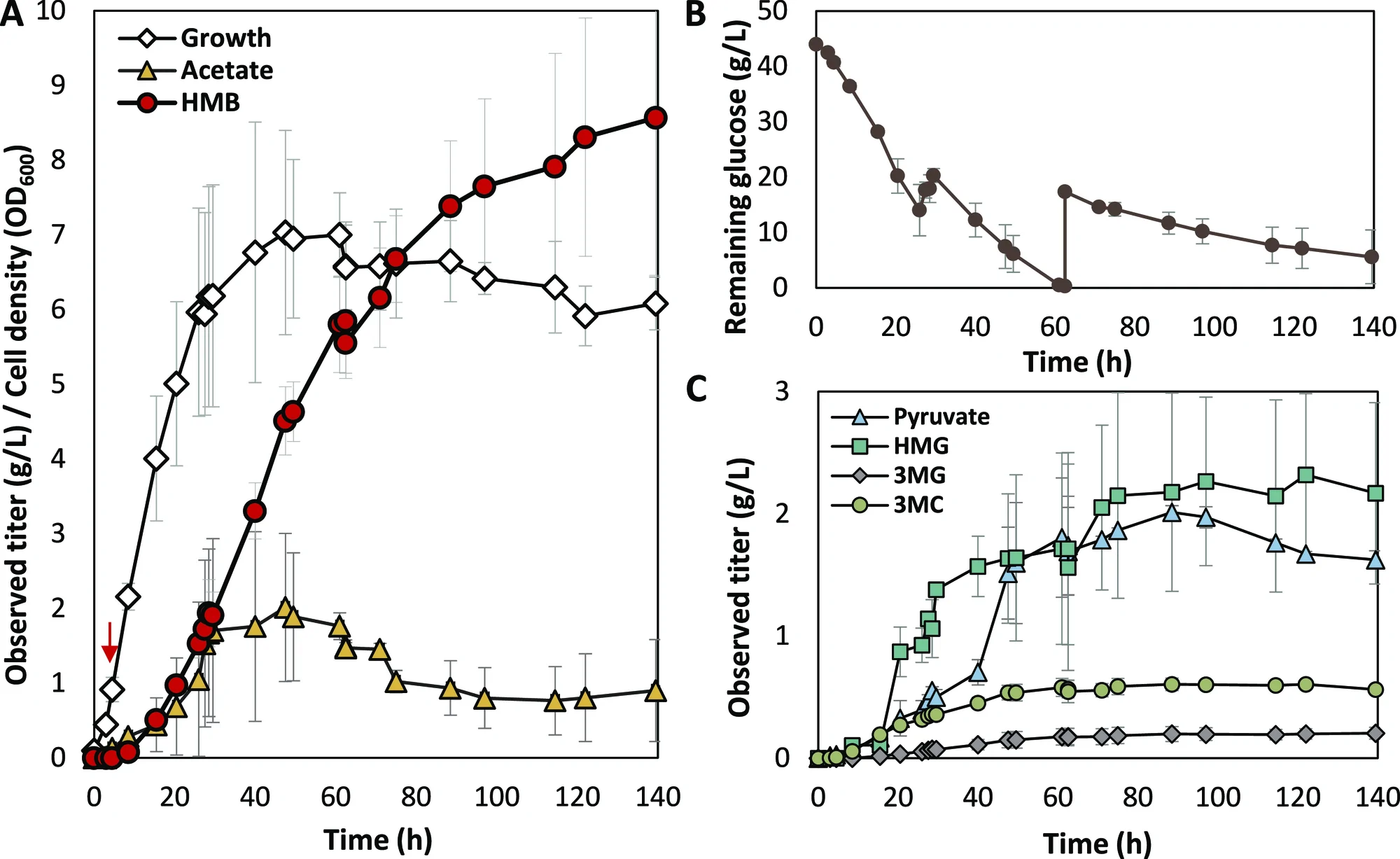
Fed-batch HMB production using glucose as a feedstock in a benchtop bioreactor. Production profiles of HMB and its byproducts in strain TS07 (EcN ΔpoxB) harboring the HMB pathway plasmid pML98 with B5 feeding with 139.5 h fed-batch fermentation. (A) Concentration of HMB, acetate and cell growth. (B) Remaining glucose concentration. (C) Concentration of pathway intermediates (HMG, 3MG, and 3MC) and pyruvate. Protein expression was induced by the addition of 0.1 mM IPTG at 4.5 h OD_600_ = 1.0, as indicated by the red arrow. Data are presented as the mean ± standard deviation of duplicate experiments.

**Table 2:** Summary of Growth, HMB Production, and Byproduct Distribution of Engineered EcN Strains under Different Optimization Stages

	metabolites concentration observed (g/L)								
strains	HMG	3MG	3MC	HMB	acetate	pyruvate	OD_600_	glucose consumption	HMB yield (g/g)
**EcN**					2.6 ± 0.2	0.057 ± 0.01	2.6 ± 0.1	11 ± 0.3	
**EcN pML98 (Tube 24 h)**	0.24 ± 0.02	0.016 ± 0.0009	0.082 ± 0.004	0.34 ± 0.02	2.5 ± 0.02	0.079 ± 0.02	2.3 ± 0.02	8.3 ± 0.4	0.041 ± 0.004
**EcN *ΔpoxB* pML98 (Tube 24 h)**	0.28 ± 0.06	0.017 ± 0.0001	0.084 ± 0.002	0.26 ± 0.04	1.1 ± 0.1	1.2 ± 0.03	2.8 ± 0.4	6.5 ± 0.2	0.040 ± 0.005
**EcN *ΔpoxB* pML98+B5 (Tube 24 h)**	0.35 ± 0.05	0.039 ± 0.004	0.14 ± 0.004	1.4 ± 0.2	0.28 ± 0.01	0.62 ± 0.06	2.8 ± 0.2	11 ± 0.9	0.13 ± 0.007
**EcN *ΔpoxB* pML98+B5 (Bioreactor 139.5 h)**	2.2 ± 0.7	0.21 ± 0.05	0.56 ± 0.02	8.6 ± 2	1.2 ± 0.7	1.6 ± 0.07	6.1 ± 0.4	62 ± 0.6	0.14 ± 0.02

Despite this significant improvement in HMB production, a noticeable leakage of carbon flux into intermediate byproducts was still observed during the fermentation. Among these, the accumulation was predominantly led by HMG, which reached a substantial concentration of 2.2 g/L by the end of the process (Figure 5C). This predominant accumulation of HMG is likely due to the broad substrate specificity of YciA, which hydrolyzes HMG-CoA given its structural similarity to the target substrate HMB-CoA. Therefore, future efforts to enhance this platform will focus on optimizing the specificity of the thioesterase or identifying a more selective enzyme. For example, the *E. coli* thioesterase TesB has been reported to exhibit higher substrate specificity than YciA, although its lower catalytic activity currently limits its application for HMB production.^21^ Resolving this nonspecific activity will be crucial to minimize the formation of related organic acids and further improve the final product purity.

### Vitamin B5 Concentration Optimization

To further enhance the cost-effectiveness of this bioprocess, a titration experiment was conducted to determine the minimum required dosage of pantothenic acid. Building upon the finding that intracellular CoA expansion drives the downstream metabolic pull, results indicated that a minimal supplementation of merely 5 mg/L of pantothenic acid achieved the maximum HMB titer in the current system (Figure 6A). Notably, this low-dose supplementation led to a reduction in both extracellular pyruvate and acetate (Figure 6B). This provides direct evidence that the PDH-mediated bottleneck was alleviated and the acetyl-CoA overflow was prevented. Furthermore, the metabolic pull generated by the expanded CoA pool not only channeled carbon flux toward HMB biosynthesis but also accelerated the overall rate of upstream glucose utilization, improving the HMB yield from glucose (Figure 6C). The observation that higher pantothenic acid concentrations yielded no further improvements suggest that the baseline cofactor requirement for sustaining this metabolic pull in EcN is potentially relatively low. Alternatively, the system may have encountered enzymatic kinetic bottlenecks. For instance, the endogenous pantothenate kinase (CoaA) could be restricted by feedback inhibition from the expanded CoA pool,^44^ or the pyruvate dehydrogenase (PDH) complex may have simply reached its maximum catalytic capacity in utilizing the available CoA. Further investigation of these potential enzymatic bottlenecks will be important for improving CoA availability and further enhancing HMB production in future studies. Ultimately, considering the comparable prices of pantothenic acid and HMB (∼$5.90 USD/kg) alongside inexpensive glucose feedstock (∼$0.60 USD/kg), this 5 mg/L low-dose supplementation successfully resolved the host-specific bottleneck while minimizing operational costs, thereby establishing a cost-effective and viable platform for HMB production.

**Figure 6:**
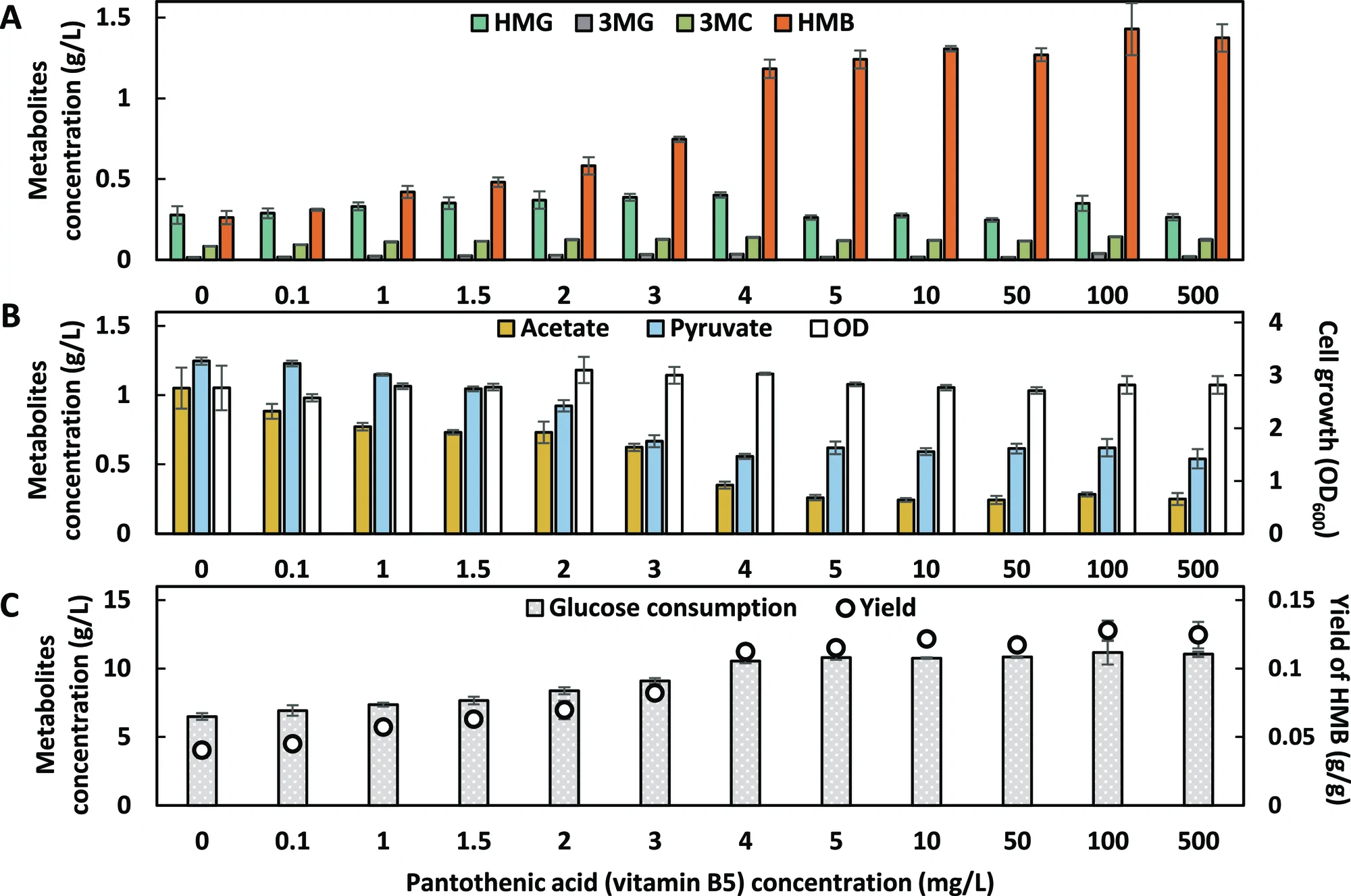
Optimization of HMB production through Vitamin B5 concentration titration. Production profiles of HMB and its byproducts in strain TS07 (EcN ΔpoxB) harboring the HMB pathway plasmid pML98 with different B5 concentration after 24 h. (A) Titers of HMB and pathway intermediates (HMG, 3MG, and 3MC) and (B) concentrations of major byproducts (acetate and pyruvate) and cell growth (C) Glucose consumption and the yield of HMB from glucose. Data are presented as the mean ± standard deviation of triplicate experiments (*n* = 3).

In summary, to our knowledge, this study represents the first implementation of a synthetic *de novo* HMB biosynthetic pathway in a probiotic host. By leveraging the low-endotoxin profile of EcN, this work establishes a solid foundation for a bioprocess for high-value supplement manufacturing. To further enhance the clinical and commercial viability of this system, future efforts will focus on transitioning from antibiotic selection and chemical induction toward antibiotic-free and inducer-free expression systems.^38,45–48^ Recent studies have demonstrated successful developments in this area, and repurposing EcN’s endogenous cryptic plasmids (e.g., pMUT1/2) combined with constitutive promoters provides a highly promising reference for our future strain optimization.^32^ Furthermore, future studies should explore engineering endogenous pantothenic acid biosynthesis to eliminate the need for exogenous supplementation.^39^ Ultimately, this work provides a foundation for developing engineered EcN as a living factory for the sustainable and safe management of muscle-wasting disorders.

## Abbreviations Used

EcN: *Escherichia coli* Nissle 1917
3MC: 3-methylcrotonate
3-MC-CoA: 3-methylcrotonyl-CoA
3MG: 3-methylglutaconate
3-MG-CoA: 3-methylglutaconyl-CoA
HMG: 3-hydroxymethylglutarate
HMG-CoA: 3-hydroxymethylglutaryl-CoA
HMB: 3-hydroxy-3-methylbutyrate
HMB-CoA: 3-hydroxy-3-methylbutyryl-CoA
Pta: phosphotransacetylase
AckA: acetate kinase
PDH: pyruvate dehydrogenase
PoxB: pyruvate oxidase
Acs: acetyl-CoA synthetase
TPP: thiamine pyrophosphate
